# Supplementary material for: Type III Secretion System-Mediated Induction of Systemic Resistance by Pseudomonas marginalis ORh26 Enhances Sugar Beet Defence Against Pseudomonas syringae pv. aptata
Source: Plants (Basel). 2025 May 26;14(11):1621. doi: 10.3390/plants14111621 (PMC12157919; doi:10.3390/plants14111621)
Supplement: Supplementary file 1 [file plants-14-01621-s001.zip › Supplementary materials.pdf]

Supplementary Materials

Supplementary Table S1. Analysis of housekeeping genes for *P. marginalis* ORh26. Each gene was analysed against the NCBI database using the NCBI BLAST tool. The table lists the species and strain with the best match, along with the sequence coverage and identity values for each alignment.

Gene	Species	Strain	Coverage	Identity
16S rRNA	*P. marginalis*	MGMM3	99%	96.77%
gyrB	*P. marginalis*	MGMM3	100%	99.96%
rpoD	*P. marginalis*	MGMM3	100%	99.68%
rpoB	*P. azotoformans*	P45A	100%	99.79%
	P. marginalis	MGMM3	100%	99.50%
recA	*P. marginalis*	MGMM3	100%	99.91%
fadI	*P. marginalis*	MGMM3	100%	99.69%
atpD	*P. marginalis*	MGMM3	100%	100.00%
	P. azotoformans	P45A	100%	100.00%
oprF	*P. marginalis*	MGMM3	100%	100.00%

Supplementary Table S2. dDDH comparison of ORh26 with type strains from TYGS. Digital DNA–DNA hybridization values (d0, d4, d6) and G+C content differences are shown for each reference strain.

Reference strain	dDDH (d0)	dDDH (d4)	dDDH (d6)	G+C difference (%)
Pseudomonas marginalis DSM 13124	74.9%	69.5%	76.5%	0.29
Pseudomonas grimontii DSM 17515	65.8%	53.9%	65.0%	0.85
Pseudomonas sivasensis P7	61.1%	37.8%	55.8%	1.43
Pseudomonas cyclaminis MAFF 301449T	62.0%	37.5%	56.3%	0.66
Pseudomonas petroselini MAFF 311094	61.0%	36.6%	55.2%	0.85

Supplementary Table S3. Locus tags and positions of genes in the T3SS operon of *P. marginalis* ORh26. Where available, similarity to proteins in *P. marginalis* SBW25 and *P. syringae* pv. *tomato* DC3000 is indicated. The proteins were annotated with the PGAP pipeline. For genes without locus tags, annotation was performed manually based on sequence homology. In locus localisation, c denotes the complementary DNA strand in the genomic sequence.

Protein	Locus tag	Locus location	Protein identity (%)	
			SBW25	DC3000
HrcW	ACKCUY_RS04760	c(993953..995017)	68.11	30.49
HrcV	ACKCUY_RS04755	c(991863..993956)		61.54
HrcD	ACKCUY_RS04750	c(990929..991828)		36.83
HrcN	ACKCUY_RS04745	c(989574..990932)		74.62
HrcO	ACKCUY_RS04740	c(989159..989587)	85.21	28.21
HrpP	ACKCUY_RS04735	c(988704..989162)	71.05	28.95
HrcQ	ACKCUY_RS04730	c(987694..988707)	84.27	
HrcR	ACKCUY_RS04725	c(987044..987697)	93.55	68
HrcS	ACKCUY_RS04720	c(986773..987036)	95.4	97
HrcT	ACKCUY_RS04715	c(985974..986762)	89.92	54.88
HrcU	ACKCUY_RS04710	c(984880..985977)	88.82	56.83
HrpV	ACKCUY_RS04705	984529..984879	72.41	29.09
HrpT	ACKCUY_RS04700	984314..984517	89.55	40.82
HrcC	ACKCUY_RS04695	982182..984317	85.55	44.41
HrpG	ACKCUY_RS04690	981775..982167	49.23	
HrcL	ACKCUY_RS04680	980888..981460	44.81	31.06
HrpD		980308..980910	49.71	31.82
HrcJ	ACKCUY_RS04675	979515..980315	77.42	60.00
HrpB		979141..979509	38.00	28.07
hp		978821..979024		

Supplementary Table S4. Detection of secretion systems in *P. marginalis* ORh26 using MacSyFinder. The system completeness score (0–1) reflects how complete the detected system is, with 1.0 indicating all mandatory components are present. The hit score represents the strength of the match between the protein and its HMM profile, while the independent e-value indicates the statistical significance of that match. Profile coverage refers to the proportion of the HMM profile covered by the hit, and sequence coverage indicates the proportion of the protein sequence aligning with the profile.

Secretion System Type	System Completeness Score (0-1)	System occurrence	Gene name	Hit score	Independent e-value	Profile coverage	Sequence coverage
T1SS	1	3	T1SS_omf	364.60	1.60E-109	1.00	0.89
			T1SS_mfp	266.30	1.40E-79	1.00	0.79
			T1SS_abc	646.00	1.40E-194	1.00	0.69
T2SS	0.923	1	T2SS_gspF	416.90	1.70E-125	1.00	0.83
			T2SS_gspE	625.40	1.20E-188	0.98	0.85
			T2SS_gspD	214.40	2.70E-64	0.99	0.24
			T2SS_gspM	36.40	2.00E-09	0.99	0.71
			T2SS_gspL	25.70	2.90E-06	0.70	0.27
			T2SS_gspK	43.50	8.90E-12	0.97	0.20
			T2SS_gspG	106.40	2.30E-31	0.94	0.55
			T2SS_gspI	57.70	4.30E-16	1.00	0.46
			T2SS_gspC	28.90	4.30E-07	0.65	0.45
			T2SS_gspH	52.70	1.50E-14	0.92	0.40
			T2SS_gspJ	55.60	1.90E-15	0.71	0.32
T3SS	1	1	T3SS_sctJ	253.70	4.80E-76	0.94	0.91
			T3SS_sctC	389.70	5.90E-117	0.99	0.72
			T3SS_sctU	355.60	8.90E-107	1.00	0.93
			T3SS_sctT	203.30	1.40E-60	0.93	0.90
			T3SS_sctS	102.60	3.00E-30	0.95	0.89
			T3SS_sctR	272.70	6.20E-82	1.00	0.92
			T3SS_sctQ	58.60	1.90E-16	0.99	0.20
			T3SS_sctN	607.80	3.30E-183	0.99	0.96
			T3SS_sctV	805.20	1.60E-242	1.00	0.96
T4SS	0.765	1	T4aP_pilE	61.50	2.80E-17	0.88	0.42
			T4aP_pilC	403.10	2.70E-121	1.00	0.83
			T4aP_pilD	274.70	1.70E-82	1.00	0.83
			T4aP_pilA	55.00	2.60E-15	0.86	0.49
			T4aP_pilX	28.50	4.00E-07	0.86	0.31
			T4aP_pilW	44.90	3.90E-12	0.51	0.26
			T4aP_pilV	56.80	6.30E-16	0.98	0.34
			T4aP_fimT	57.50	4.40E-16	0.96	0.33
			T4aP_pilQ	198.40	2.00E-59	0.93	0.36
			T4aP_pilO	80.00	5.60E-23	0.99	0.46
			T4aP_pilN	101.60	1.30E-29	0.99	0.78
			T4aP_pilM	141.80	6.60E-42	1.00	0.75
			T4aP_pilT	536.20	9.80E-162	0.99	0.99
T5SS	1	1	T5aSS_PF03797	208.00	6.50E-62	1.00	0.32

			T5bSS_translocator	112.50	6.60E-33	0.90	0.90
T6SS	0.857	1	T6SSi_tssI	698.70	2.10E-210	0.94	0.87
			T6SSi_tssH	1376.10	0.00E+00	1.00	0.99
			T6SSi_tssG	372.40	5.90E-112	0.99	0.94
			T6SSi_tssF	751.30	2.50E-226	1.00	0.99
			T6SSi_tssE	148.00	4.90E-44	0.99	0.88
			T6SSi_tssD	186.10	1.60E-55	1.00	0.98
			T6SSi_tssC	752.60	5.40E-227	0.99	0.97
			T6SSi_tssB	219.80	5.10E-66	0.98	0.95
			T6SSi_tssJ	115.40	5.20E-34	0.94	0.86
			T6SSi_tssK	567.20	8.00E-171	1.00	0.99
			T6SSi_tssL	247.60	3.30E-74	1.00	0.50
			T6SSi_tssM	1209.40	0.00E+00	0.99	0.94

Supplementary Table S5. Results of the Effectidor search for T3Es in the genome of *P. marginalis* ORh26 and SBW25. Genes with a score greater than 0.3 are shown. The genome ORFs represent the accession numbers of the genes. The functional annotations were created with SMART. To view the dataset, please open the Supplemetary Table S5.xlsx file.

Supplementary Table S6. Primers used in this study.

Primer	Sequence	Target	Reference
IA089	aaaaaatctagaCCGACGATCAGGTCCTGCAGCAG	600 bp fragment of *hrcT*	This study
IA090	aaaaaactcgagTGTGATGGTCATGGCATTGCTGCC		
IA095	CTCATGCCATTAGGCGCAGG	The region surrounding *hrcT*	
IA096	CTGCTGTTGGTCATCTGCACATC		
NPR1BvF	TCATGAAGCTTGTCGTCCTG	Expression of *npr1* via qPCR	[13]
NPR1BvR	ATACACCTTGCCAGCAATCC		
25RNK_F	AGACAAGAAGGGGCAACGAG	Expression of 25S rRNA via qPCR	[14]
25RNK_R	CACATTGGACGGGGCTTTTC		
BvNCED-F	AGGAACAAACTGGGGAGGAAAA	Expression of *nced* via qPCR	[15]
BvNCED-R	TCCCAATTGATCTCATCGTGC		
BvLOX-F	ATCGGCAGTTGAGTGCAATG	Expression of *lox* via qPCR	
BvLOX-R	CTGCCATTCCCCTGCTTACA		
BvMYC2-F	AGTGAGCTTGACGTGCAGTA	Expression of *myc2* via qPCR	
BvMYC2-R	CCCCTCATCTGCCTCAAGAAATAC		
Psy_F	ATGATCGGAGCGGACAAG	Quantification of *P. syrinagae* P21	[16]
Psy_R	GCTCTTGAGGCAAGCACT		
IA067	CCATGACCATCACAATGGTGTGC	Quantification of *P. marginalis* ORh26	This study
IA068	CTGCCCAGCCTCTTGATCG		

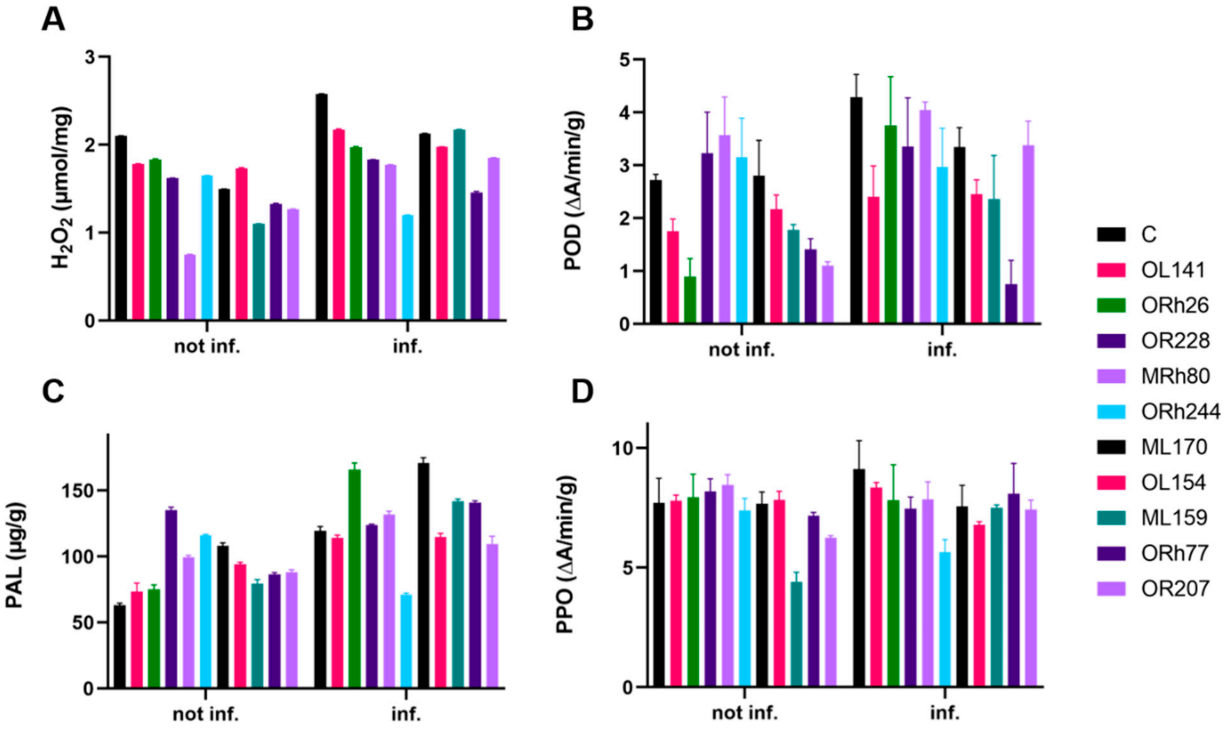

Supplementary Figure S1. H_2O_2 content and enzyme activity in sugar beet plants treated with different T3SS-positive *Pseudomonas* isolates. The values for infected (inf.) and non-infected (not inf.) plants are shown. **A -** Changes in H_2O_2 content. **B -** Peroxidase (POD) activity. **C -** Phenylalanine ammonia lyase (PAL) activity. **D -** Polyphenol oxidase (PPO) activity.

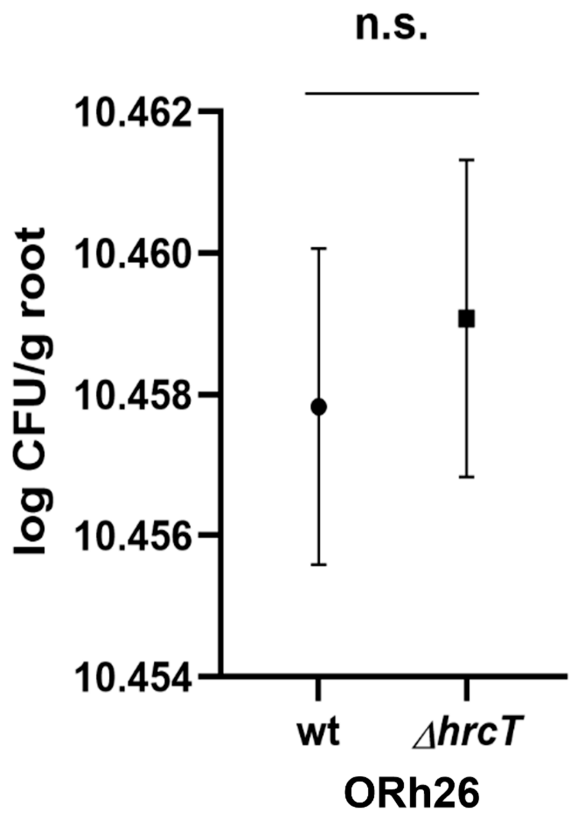

Supplementary Figure S2. Root colonization by *P. marginalis* ORh26 wt and ΔT3SS mutant (Δ*hrcT*) strains on sugar beet. Colony-forming units (CFUs) were quantified per gram of root tissue at 7 days post-infection, in plants infected with *P. syringae* P21. No significant difference in colonization efficiency was observed between the wt and mutant strains at the time of lesion assessment.

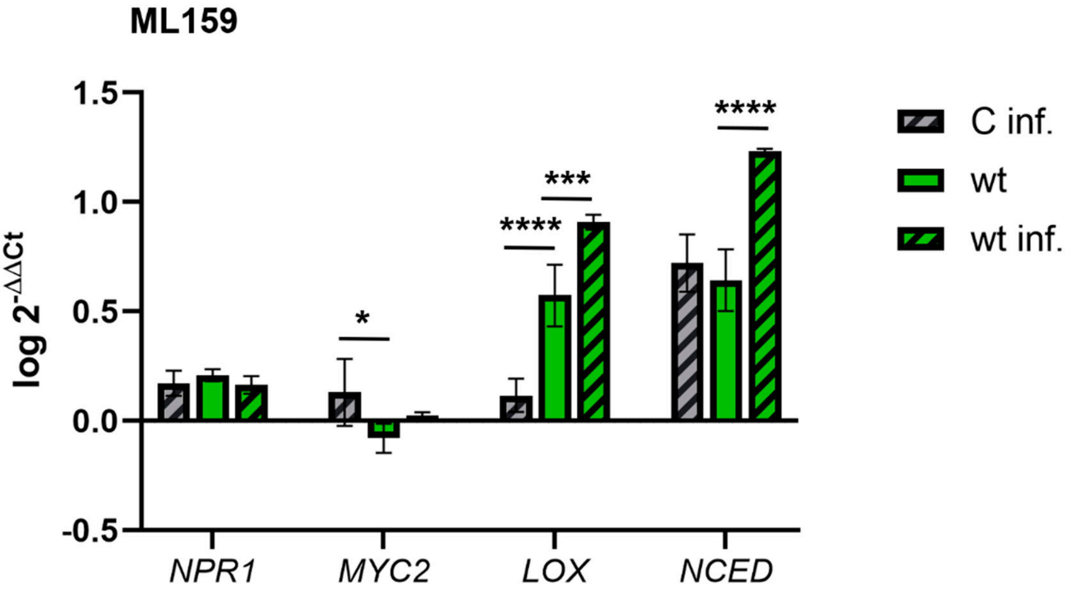

Supplementary Figure S3. Immune gene expression levels in sugar beet treated with *P. lurida* ML159. Relative gene expression was analysed in uninfected and infected (inf.) plants. Expression levels were normalised to the 25S rRNA housekeeping gene, with the uninfected and untreated control serving as the reference group. Significant differences are indicated as follows: $p < 0.05$ (*), $p < 0.01$ (**), $p < 0.001$ (***), and $p < 0.0001$ (****). ns means no significant difference.
